# “Nano in Nano”—Incorporation of ZnO Nanoparticles into Cellulose Acetate–Poly(Ethylene Oxide) Composite Nanofibers Using Solution Blow Spinning

**DOI:** 10.3390/polym16030341

**Published:** 2024-01-26

**Authors:** Caroline Voorhis, Javier González-Benito, Ana Kramar

**Affiliations:** 1 School of Science, Marist College, 3399 North Road, Poughkeepsie, NY 12601, USA; caroline.voorhis1@marist.edu; 2 Department of Materials Science and Engineering and Chemical Engineering, Institute of Chemistry and Materials Álvaro Alonso Barba, IQMAAB, Universidad Carlos III de Madrid, Avda. Universidad 30, 28911 Leganés, Spain; javid@ing.uc3m.es; 3 Novel Materials and Nanotechnology Group, Institute of Agrochemistry and Food Technology (IATA), Spanish Council for Scientific Research (CSIC), Calle Catedrático Agustín Escardino Benlloch 7, 46980 Paterna, Spain

**Keywords:** solution blow spinning, cellulose acetate, zinc oxide, nanofibers, nanoparticles, poly(ethylene oxide)

## Abstract

In this work, the preparation and characterization of composites from cellulose acetate (CA)–poly(ethylene oxide) (PEO) nanofibers (NFs) with incorporated zinc oxide nanoparticles (ZnO-NPs) using solution blow spinning (SBS) is reported. CA–PEO nanofibers were produced by spinning solution that contained a higher CA-to-PEO ratio and lower (equal) CA-to-PEO ratio. Nanoparticles were added to comprise 2.5% and 5% of the solution, calculated on the weight of the polymers. To have better control of the SBS processing conditions, characterization of the spinning suspensions is carried out, which reveals a decrease in viscosity (two- to eightfold) upon the addition of NPs. It is observed that this variation of viscosity does not significantly affect the mean diameters of nanofibers, but does affect the mode of the nanofibers’ size distribution, whereby lower viscosity provides thinner fibers. FESEM–EDS confirms ZnO NP encapsulation into nanofibers, specifically into the CA component based on UV-vis studies, since the release of ZnO is not detected for up to 5 days in deionized water, despite the significant swelling of the material and accompanied dissolution of water-soluble PEO. Upon the dissolution of CA nanofibers into acetone, immediate release of ZnO is detected, both visually and by spectrometer. ATR–FTIR studies reveal interaction of ZnO with the CA component of composite nanofibers. As ZnO nanoparticles are known for their bioactivity, it can be concluded that these CA–PEO–ZnO composites are good candidates to be used in filtration membranes, with no loss of incorporated ZnO NPs or their release into an environment.

## 1. Introduction

Nanofibers (NF) and nanoparticles (NP) are two types of nanomaterials that have attracted much attention in recent years due to their unique properties and potential applications in various fields, including the biomedical, environmental, and energy sectors [1]. When nanofibers and nanoparticles are combined, they can form a new class of nanocomposites that exhibit enhanced properties compared to the individual components. Nanofibers can provide a high surface area-to-volume ratio and porosity to the final material, while nanoparticles can impart unique optical, magnetic, antimicrobial or catalytic properties [1,2]. Embedding nanoparticles in nanofibers can be achieved by various methods, the most popular being electrospinning. In the electrospinning method, a liquid containing a polymer system and nanoparticles is spun, using a voltage differential, to obtain a fibrous material that is a nanofiber–nanoparticle (NF/NP) composite [2,3,4]. However, other NF–NP composites can be obtained using surface deposition/adsorption by depositing nanoparticles on the previously spun fibers [2,5]. Nanofibers combined with nanoparticles have shown promising applications in various fields. For instance, they can be used as nanofiltration and pollutant degradation membranes [6,7,8,9], wound dressings [10] or drug delivery systems [11], among many others. In these applications, the combination of nanofibers and nanoparticles can provide improved mechanical strength, biocompatibility, and enhanced functionality.

Apart from the above, special interest lies in using natural polymers in the form of nanofibers, as they are biodegradable, biocompatible, and non-toxic [12,13,14]. Cellulose, the most abundant natural polymer, and its derivatives have been the focus of research due to their biocompatible nature. The conversion of cellulose and its derivatives into nanofibers has been the subject of many studies in the last few decades [15,16,17,18,19,20,21]. Cellulose acetate, a thermoplastic derivative of cellulose, is especially notable as a versatile derivative. It is soluble in many organic solvents, with the possibility to be processed in many forms, including film, fibers, and nanofibers [18,22,23]. Doping of cellulose acetate with nanoparticles can lead to the development of versatile antimicrobial material if the added nanoparticles possess some antimicrobial or bioactive effect. Such nanoparticles are mostly based on silver, copper, titanium dioxide or zinc oxide [24,25,26,27]. Zinc oxide NPs (ZnO NP) have many potential applications: with their photocatalytic activity [28], they can be used for water disinfection [29,30] and in biomedical applications as an antimicrobial additive to obtain medical textile materials [31]. The most common techniques for incorporation of nanoparticles into CA include solution casting [25,32,33] or electrospinning (ES) [26,34,35,36].

Here, we report a novel method, solution blow spinning, to incorporate zinc oxide nanoparticles (ZnO NPs) into nanofibers prepared fromcellulose acetate–polyethylene oxide (CA–PEO) mixture. Solution blow spinning (SBS) is a method for nanofiber preparation, and to the best of our knowledge, this is the first report of nanoparticles incorporated into cellulose acetate nanofibers using SBS. This technique has several advantages over other nanofiber fabrication techniques [37,38]. It is a simple and versatile technique that does not require a high-voltage power supply or complex equipment. Additionally, SBS can produce high-quality nanofibers at a higher rate than electrospinning. Finally, SBS can be used in situ directly onto the target, for example, onto some object that needs to be covered with fibers/films, including human skin [37].

In this paper, we present incorporation of ZnO nanoparticles into cellulose acetate–poly(ethylene oxide) CA–PEO nanofibers. The use of PEO during spinning plays a supporting role in CA spinning [39], since CA is difficult to process using SBS, because of the inability to obtain bead-free nanofibers [40]. Both polymers, CA and PEO, are biodegradable and biocompatible, which makes them suitable for potential biomedical applications, especially considering the bioactivity of ZnO [31]. Finally, with the objective of making comparisons of morphology and molecular interactions, two nanocomposite systems that contain an equal composition of CA (12%) were prepared by SBS and ES, with the difference being that in SBS, the spinning aid PEO was used. The purpose of this is to look for more information that allows for better decision-making when deciding the best processing method to prepare nanofibers for a particular purpose.

In addition, we compare the morphology and molecular interactions between nanofibers obtained by SBS of CA 12% with addition of PEO and ZnO NPs and by ES of pure CA 12% with ZnO.

## 2. Materials and Methods

### 2.1. Materials

Poly(ethylene oxide) (PEO; Mv 100,000), cellulose acetate CA (Mn 30,000), acetone (HPLC > 99.9%), and chloroform (anhydrous, ≥99%) were supplied by Sigma-Aldrich Merck (St. Louis, MO, USA)and used as received without further purification. Commercial zinc oxide (ZnO) nanopowder from Sigma-Aldrich Merck (St. Louis, MO, USA), with mean diameter of particles ≤100 nm, was used; according to the certificate of analysis (COA) of this batch, the nanopowder had an average particle size of 67 nm. Complete characterization of ZnO NPs can be found in the literature [41].

### 2.2. Preparation of Polymer Solution and Suspensions

**Figure 1:**
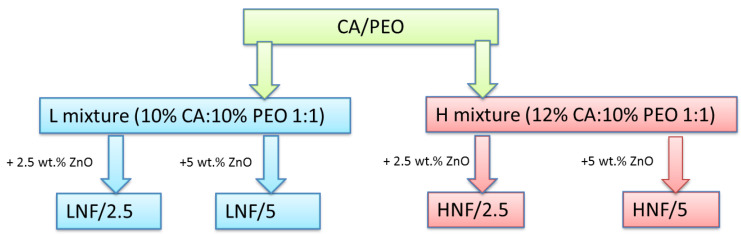
Scheme of samples and their corresponding sample marks prepared in this work using solution blow spinning.

Polymers were dissolved separately 24 h before spinning following a protocol that can be found in a previous study [39]. CA was dissolved in acetone and PEO in a mixture of acetone and chloroform. After that, an equal volume of each polymer solution was mixed (1:1 ratio) to prepare the corresponding solutions of the two polymers, and after 1 h, the required amount of ZnO nanoparticles was added to the solution, followed by 10 min treatment in ultrasonic bath, to prepare the corresponding suspensions. After the ultrasonic bath, samples were left to stir on a magnetic stirrer for 30 min prior to spinning. LNF denotes the mixtures prepared by using a 10% *w*/*v* solution of CA and a 10% *w*/*v* solution of PEO, and HNF denotes mixtures prepared by using a 12% *w*/*v* solution of CA and a 10% *w*/*v* solution of PEO in equal ratio (Scheme in Figure 1). ZnO NPs were added in concentrations of 2.5 wt% and 5 wt%, calculated on the total weight of polymer mixture in the solution.

### 2.3. Solution Blow Spinning (SBS) and Electrospinning (ES)

Solution blow spinning was performed using a homemade device at UC3M as reported previously [39,40]. The distance from the tip of the needle to the collector (working distance) was set to 15 cm, the rotational speed of the collector was 250 rpm, the pressure of the propellent air was 1.5 bar for the LNF mixtures and 2 bar for the HNF mixtures, and the injection rate (IR) was set at 0.25 mL/min. The materials were collected on the aluminum foil used to cover the rotating cylindrical collector.

Electrospinning was performed on the commercial device Spinbox (Bioinicia, Paterna, Spain), under the following spinning parameters: IR 30 µL/min, RPM 250, working distance 10 cm, voltage 25 kV. In this case, a solution of pure CA 12% *w*/*v* in acetone was used (without PEO), whereby the same concentrations of ZnO were considered (2.5 wt.% and 5.0 wt.%), as in the case of SBS.

### 2.4. Characterization

#### 2.4.1. Viscosity of Spinning Solutions

Haake Viscotester IQ: parallel round-plate configuration, gap between the plates 1 mm, step increase of shear rate, 100 points measured per shear rate segment of 30 s duration. Measurements were performed at 25 °C. Shear rates were used in a range of 100–900 s^−1^.

#### 2.4.2. Morphology of Samples Using Field-Emission Scanning Electron Microscopy Coupled with Energy Dispersive X-ray Spectroscopy (FESEM–EDS)

Morphology was studied using the field-emission scanning electron microscope (FESEM) TENEO-FEI. An ETD detector was used to generate images of the samples from the signal arising from secondary electrons (SEs), which mainly yields morphological information. On the other hand, a CBS (circular backscatter) detector was also used in this case to generate images from the signal arising from backscattered electrons, which, apart from giving morphological information, shows contrast associated with compositional information necessary to distinguish the polymers from the ZnO NPs. Energy-dispersive spectroscopy (EDS) was used to carry out elemental microanalysis on the surface of the materials prepared. In every case, an acceleration voltage of 10 kV was used. Prior to imaging, samples were gold-coated for 25 s by sputtering using a Leica EM ACE200 low-vacuum coater.

Analysis of images was conducted using ImageJ 153 open access software. For determination of the average fiber diameter, between 80 and 150 measurements were taken into analysis, while for nanoparticles, the longest axis length of irregularly shaped aggregates was presented as the size of aggregates, and due to limited amounts, 15–30 measurements per sample containing ZnO were performed.

#### 2.4.3. Structure and Molecular Interactions Using Infrared Spectrometry: ATR–FTIR

An FTIR spectrometer (Thermo Fisher Nicolet iS5 with an ATR device (diamond window, GladiATR PIKE Technologies, Thermo scientific, Thermofisher, Madison, WI, USA) was used for structural characterization of the samples. Measurements were performed in a range of 400–4000 cm^−1^ using 32 scans and 4 cm^−1^ resolution.

#### 2.4.4. Release of ZnO Using UV-Vis Spectrometry

Release of nanoparticles was studied by absorption using UV-vis spectroscopy on a Jasco V650 UV-vis spectrophotometer (Jasco, Madrid, Spain). Nanofibrous membranes (approx. 45 mg) of CA–PEO (with and without ZnO) were put in a glass bottle with deionized water (25 mL) and occasionally shaken. A small amount of liquid in which the NPs from the materials are expected to be released was taken from a bottle at increasing time intervals (5, 10, 30 min) up to 1 h, at 24 h and at 120 h (5 days), and its absorbance was measured in a range of 250 to 650 nm to detect the signals potentially originating from the released ZnO. Apart from the tests with the materials, using the same spectrometer, pure ZnO suspensions in water were measured to determine the sensitivity of the device. The lowest absorbance signal detected for ZnO nanoparticles corresponds to their concentration in water of 0.003 mg/mL.

## 3. Results and Discussion

### 3.1. Morphological Analysis of Prepared Nanocomposites

Preparation of materials by solution blow spinning requires optimization of the process from a careful analysis of morphology [42]. Many of the material properties depend on the size of nanofibers, including their diameters and interconnections; therefore, good control of the morphology should be ensured.

**Figure 2:**
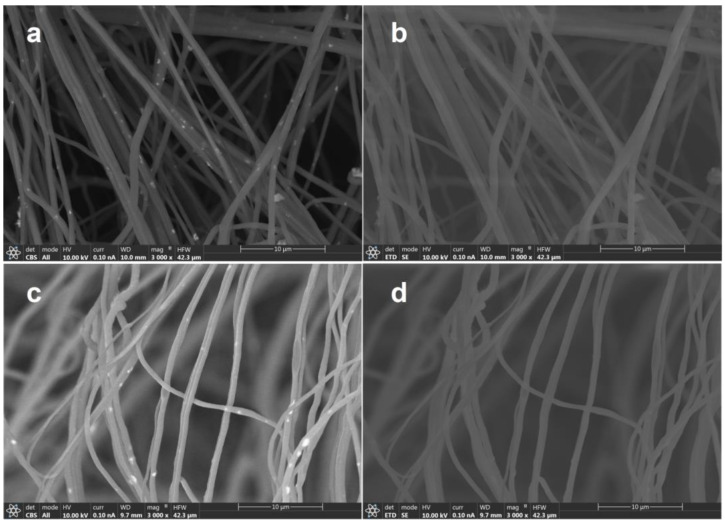
FESEM images obtained by different detectors of HNF samples with 2.5% ZnO NP (**a**) CBS detector and (**b**) ETD detector, and HNF samples with 5% ZnO NP (**c**) CBS detector and (**d**) ETD detector.

Figure 2 shows FESEM images obtained by different detectors of HNF samples with 2.5% and 5% ZnO NPs. Materials made by thin fibers of less than 700 nm of diameter were obtained (Figure 2).

When studying the organic–inorganic composite, especially in this work, and using organic polymers as matrix and inorganic nanoparticles as filler, special attention must be given to the detector used during scanning electron microscopy. The materials prepared in this work are obvious confirmation of this nuance, since by using a classical EDT detector, it is almost impossible to differentiate the nanofibers and nanoparticles (Figure 2b,d). Particularly, as in this study, where NPs are expected to be inside nanofibers, it becomes obvious that CBS or backscattered electron collection is crucial for obtaining proper images (Figure 2a,c) and consequently their analysis. Apart from the morphology, intensity of backscattered electrons depends on the elements that form part of the material, giving images with contrast on those regions with different elemental composition. Figure 2a,c show bright spots that are quite uniformly dispersed along the fibers, which are indicative of the presence of a Zn-heavier element. As highlighted earlier, currently there are no available reports regarding the solution blow spinning of CA–PEO with ZnO or the SBS of pure CA with ZnO.

**Figure 3:**
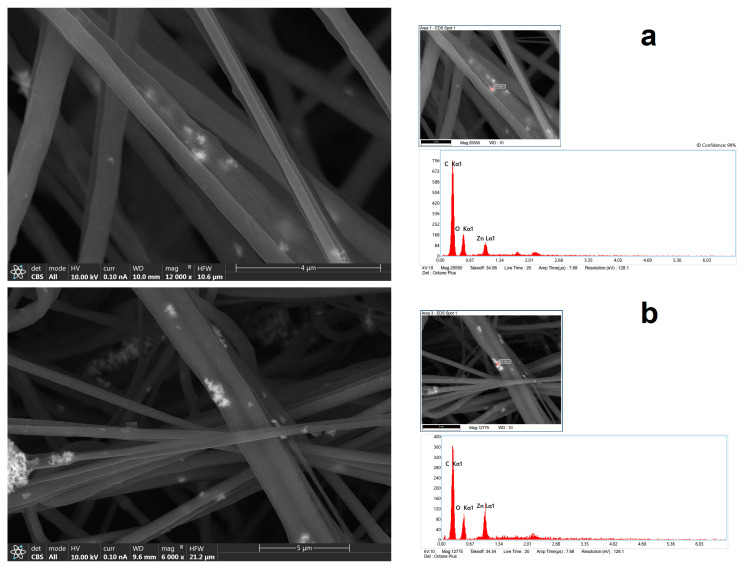
FESEM images with CBS detector coupled with EDS analysis of chemical composition of nanofiber (**a**) HNF with 2.5% ZnO and (**b**) HNF with 5% ZnO.

In Figure 3, by using a CBS detector, it was possible to see NPs—or more precisely, their aggregates inside nanofibers—at a higher magnification. Using EDX analysis on the bright spots, it was possible to confirm the presence of Zn. However, the peak in the X-ray spectrum associated with the Zn (Figure 3b) is not as intense or of higher intensity than those corresponding to C and O (specifically, the C arises from polymer(s) themselves), which may mean that the bulk electron beam–material interaction includes all materials of the composites, ZnO, and polymers.

**Figure 4:**
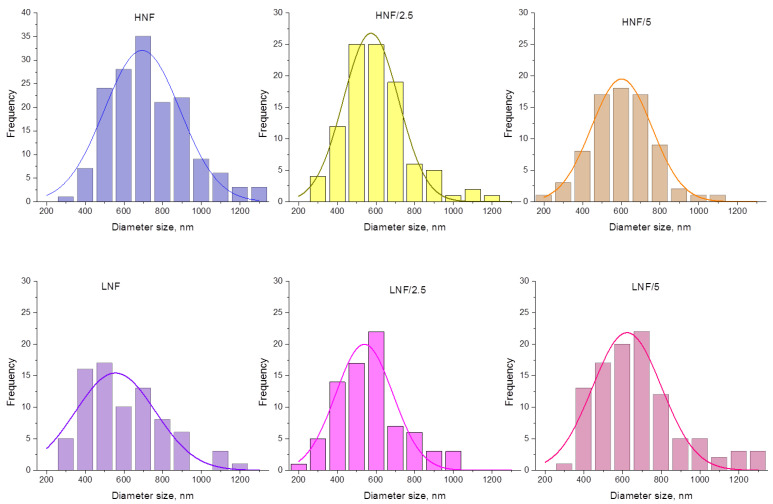
Size distribution analysis and Gaussian curve fitting of experimental data for all the samples in this study.

The size distribution of nanofibrous composites with ZnO is given in Figure 4. All distributions can be fitted by Gaussian normal distribution function, and when lower concentration of CA is used (equal ratio) in the CA–PEO composite (samples LNF), a wider and lower peak of Gaussian distribution appears, compared to HNF (approx. for LNF around 550 nm vs. HNF around 690 nm). Upon addition of 2.5% ZnO (sample LNF/2.5), more thin fibers appear in the distribution (fibers below 250 nm, in a range 150–250) and the Gaussian peak is slightly lower, but when 5% ZnO is added, the Gaussian peak is shifted toward higher values. In samples prepared using higher CA-to-PEO ratio (HNF), it is the opposite: the peak of Gaussian shifts, in both cases, to the left, i.e., lower values of diameters, and again upon the addition of 2.5% is lower compared to samples with added 5% ZnO. This can be better visualized if we directly compare Gaussian curves obtained for all samples before and after the addition of ZnO (Figure S1).

**Figure 5:**
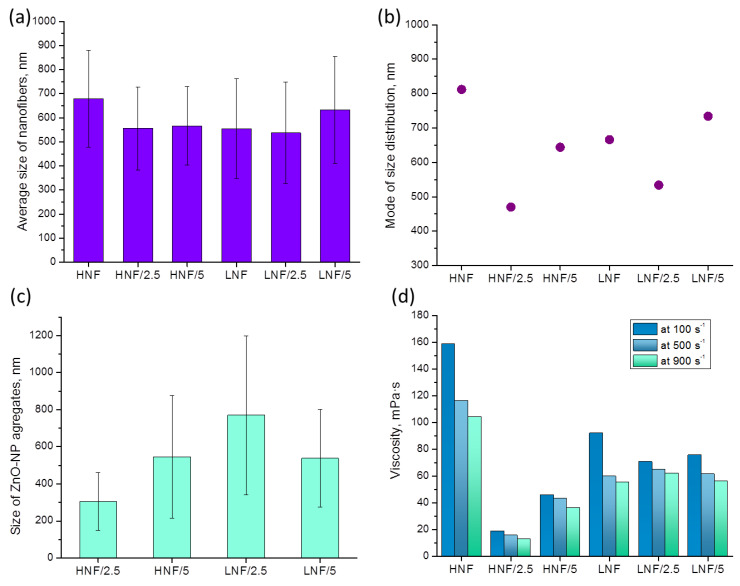
Values of mean (**a**) and mode (**b**) of size distribution of produced nanofibers depending on the composition and size of ZnO NP aggregates (**c**) on the formed nanofibers obtained from FESEM images using ImageJ software; apparent viscosity (**d**) at different shear rates of polymer-NP composite solutions used to produce nanofibers using SBS.

Analysis of images obtained in FESEM revealed that the mean diameter of nanofibers (Figure 5a), considering the deviation, does not differ depending on the composition of materials. However, if we consider the mode (the most frequent value in the distribution), we can see that it significantly differs in variously composed samples (Figure 5b). The lowest mode appears in samples produced using higher amounts of CA compared with PEO and with 2.5% added ZnO, while the highest mode appears in the sample HNF without ZnO nanoparticles. If we look at the data on viscosity (Figure 5d), apart from obvious shear-thinning behavior of polymer solutions, we can see that for the two samples with the highest and the lowest apparent viscosity (Figure 5d), there is also the highest and the lowest mode of nanofiber size (Figure 5b). Although this will be the object of further studies, at this moment it can be concluded that the addition of ZnO with a strong decrease in viscosity leads to the production of thinner nanofibers. 

The complexity of size distribution analysis of nanofibers in this work comes as a consequence of two effects: first, the nanofibers without ZnO are formed in such a way that PEO envelopes the CA, which leads to the effect of several CA nanofibers being enveloped by PEO [39], and the addition of ZnO in polymer solution disrupts the CA–PEO system, which is reflected in the observed change in viscosity.

The decrease in viscosity is known to influence the nanofiber size, and the formation and morphology of nanofibers highly depend on the viscosity and entanglement of polymer chains. This is highlighted in the electrospinning process [43,44], which is explained by the fact that lower viscosity, achieved in a lower concentration of polymer solution, usually contains less entanglement of macromolecular chains, and thus effectively produces thinner fibers. Unlike in other works, we present that the change in viscosity is achieved through simple addition of nanoparticles and obviously influenced the size distribution, as seen in Figure 4, as well as the viscosity and mode of distribution given in Figure 5.

**Figure 6:**
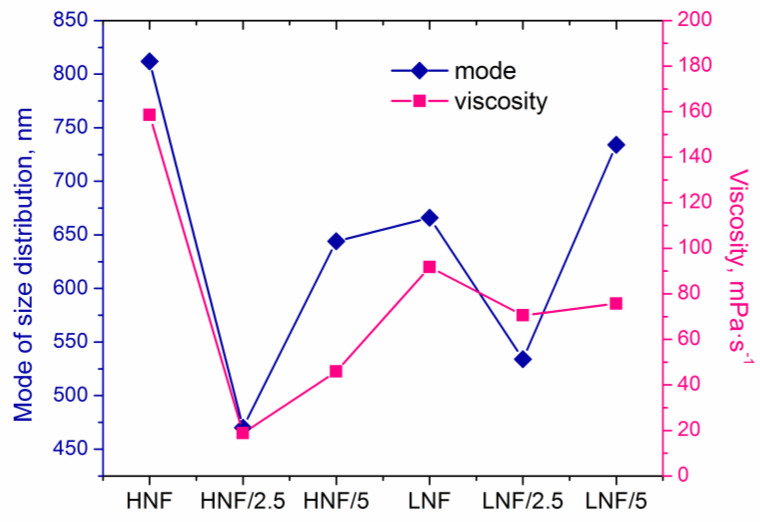
Mode of size distribution and apparent viscosity of the samples.

In Figure 6, we plotted mode of size distribution and viscosity (measured at a shear rate of 100 s^−1^) in different samples, and we can observe that the trends are almost overlapping. The only slight exception is the sample LNF/5 compared to the sample LNF/2.5, where the viscosity increased by only a small value, but the mode changed significantly.

The addition of nanoparticles to polymer solutions in some cases leads to a reduction in the viscosity of the solution [45,46]. This effect is often referred to as “nanoparticle-induced viscosity reduction” or “viscosity reduction by nanoparticles”. The exact mechanism by which nanoparticles reduce the viscosity of polymer solutions is still a subject of research, but several mechanisms have been proposed. One is that the nanoparticles disrupt the entanglement of polymer chains, which leads to a decrease in the solution viscosity by enhancing free volume in polymer solution, since it was also established that the T_g_ of the polymer, after NP addition, decreases in a study by Tuteja et al. [47]. Another hypothesis suggests that the nanoparticles are enveloped by shorter polymer macromolecules that do not directly participate in entanglement necessary for nanofiber formation; this reduces the friction between the polymer chains during the flow, which can explain the strong reduction in viscosity with shear rate [45]. A similar study conducted by Rokbani and Ajji [48] followed electrospinning of PLA with ZnO and found a decrease upon the addition of more than 3 wt% of ZnO to PLA solution, but they attributed this to accelerated polymer degradation since they measured the viscosity after 5 and 25 days. In the same study, unfortunately authors did not mention the size of the produced nanofibers or the possible effect of viscosity on the size of the nanofibers.

In another study by Chae and Kim [49] where influence of ZnO on viscosity of polystyrene (PS) and polyacrylonitrile (PAN) was studied, authors proposed that the formation of ZnO agglomerates is the reason behind decreased viscosity above critical NP concentration. As authors pointed out, “some part of the adsorbed polymer on the nanoparticles can be confined by excessive nanoparticles, leading to the reduction of the polymer concentration in the liquid phase” [49]. Therefore, for a polymer to have a reduction in its solution viscosity instead of an expected increase upon nanoparticle addition, it should have affinity towards nanoparticles, but at the same time, those nanoparticles need to have a tendency to aggregate, which is presented in our work as well.

The results from viscosity undoubtedly indicate that a strong interaction exists between CA and ZnO, more so than the interaction between PEO and ZnO.

The apparent viscosity of a pure CA solution (12% in acetone) at 100 s^−1^ shear rate is 835 mPa·s, and for 10% PEO (in acetone–chloroform mixture) is 48.5 mPa·s. The addition of PEO strongly reduces the viscosity of CA solution, as reported in literature [34], but the addition of ZnO reduces it even further. In our study, an indirect indicator of strong interaction between CA and ZnO can be the observed phenomenon that a strong reduction in viscosity occurs when ZnO is added to a solution that contains a higher amount of CA (Figure 5d). The lower viscosity is found in HNF solutions with added ZnO compared to the corresponding LNF with ZnO. Furthermore, due to a good dispersion of a small amount of ZnO, there is a lower viscosity in samples that contain lower amounts of ZnO. This is also confirmed by the smallest aggregate, (Figure 5c), even though these data should be analyzed with caution due to the small number of measurements. Further increase in the amount of ZnO leads to the formation of bigger aggregates.

The effect of nanoparticles on solution viscosity obviously depends on several factors, such as the type and size of the nanoparticles, the concentration of the nanoparticles in the solution, and the properties of the polymer itself. For example, the addition of TiO_2_ nanoparticles [50], silver nanoparticles, [51] or glass nanoparticles [52] in CA caused an increase in viscosity of the polymer solution. Nanoparticle-induced viscosity reduction will be the object of further study, since this effect has potential applications in a variety of fields, such as in the production of coatings, adhesives, and composites. By reducing the viscosity of polymer solutions, nanoparticles can make processing these materials more feasible and can also improve the materials’ properties. However, it is important to note that the addition of nanoparticles can also have other effects on the properties of polymer materials, such as changes in their optical, thermal, and mechanical properties, which must be carefully considered in the design of any nanoparticle–polymer composite.

This phenomenon of viscosity reduction due to the addition of nanoparticles can be especially important in spinning technologies, since polymer solutions of high viscosity are not suitable for nanofiber processing because of spinning interruption (e.g., blocking of the nozzle), while too low viscosities can suggest that entanglement of polymer macromolecules is too low and thus cause the production of fibers with beads or even beads only, without nanofiber formation.

### 3.2. Release Studies of ZnO Using UV-Vis

**Figure 7:**
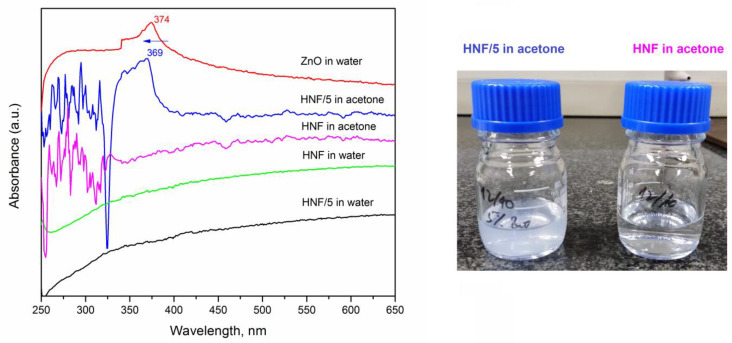
UV-vis absorbance spectra for different nanofibrous materials, without (HNF) and with 5% ZnO (HNF/5) after 5 days in water and immediately after dissolving them in acetone (**left**); photograph (**right**) of vials containing HNF/5 (translucent) and HNF (transparent) in acetone.

We studied the release kinetics of ZnO from composites in water for up to 5 days, knowing that PEO dissolves in water and that CA nanofibers have significant swelling ability [39]. The pure ZnO suspension in water was also measured, and a peak at 374 nm (Figure 7, red line) appeared, which corresponds to the values reported in the literature [53,54].

After 5 days of keeping the nanofibers in deionized water, the UV absorption peak corresponding to ZnO at around 374 nm did not appear in spectra (Figure 7, sample HNF/5 in water). Only after putting the HNF/5 material in acetone an absorption peak at 369 nm appears and the translucent system is obtained (consequence of ZnO nanoparticles release). This blue shift from 374 to 369 nm (marked by arrow in Figure 7) can be a consequence of the varying sizes of ZnO suspended in water, including the ones inside CA nanofibers. It was reported how maximum absorbance shifts depend on the size of nanoparticles [53], and aggregation of nanoparticles tends to be different when they are processed with polymer solution and with pure solvent [55].

### 3.3. Structural Characterization Using ATR–FTIR Spectroscopy

**Figure 8:**
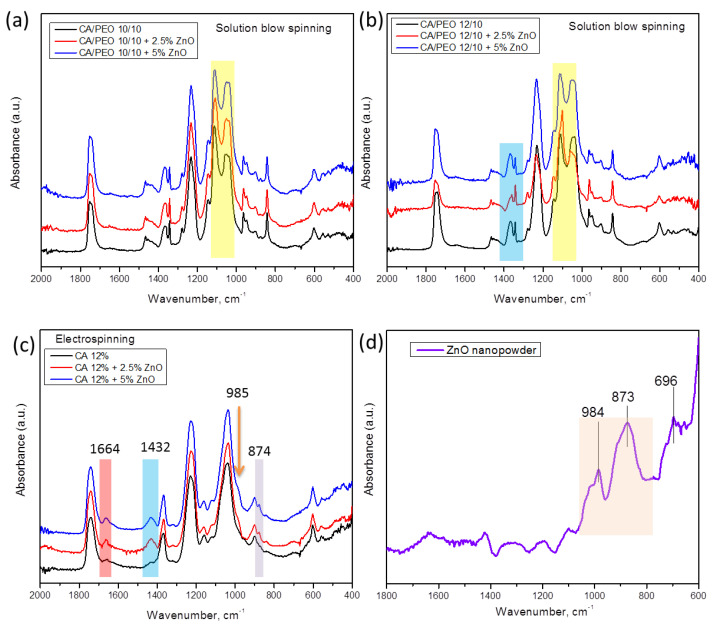
ATR-FTIR absorption spectra of samples of CA–PEO nanofibers spun with ZnO using solution blow spinning of different concentrations of CA and ZnO: (**a**,**b**), samples of pure CA nanofibers produced by electrospinning (**c**), and commercial ZnO nanopowder (**d**).

For a deeper insight into the structure and interactions between polymeric nanofibers and inorganic nanoparticles, a careful analysis of ATR–FTIR spectroscopy can be undertaken. In Figure 8a,b, ATR–FTIR spectra of the different materials prepared by SBS are shown, where the different concentrations of CA in CA–PEO composites are considered. The overall appearance of the spectra is similar to that in our previous work [39], wherein combined peaks from cellulose acetate and PEO can be seen. The most prominent difference upon the addition of ZnO to polymer mixture is found in the region between 1146 cm^−1^ and 1035 cm^−1^, in which the relative intensity of the peaks that can be observed changes upon the addition of nanoparticles (Figure 8a,b). In particular, the doublet peaks appear at approx. 1109 cm^−1^ and 1035 cm^−1^ and come from the combination of peaks originating from PEO and CA (for pure PEO, it is found at 1095 cm^−1^, and in CA–PEO composite, this peak is shifted to 1109 cm^−1^ [39]). On the other hand, the absorbance bands at 1051 cm^−1^ and 1037 cm^−1^ correspond to combined peaks of PEO (1060 cm^−1^ and 1035 cm^−1^ assigned to stretching of ether group in PEO [56]) and 1035 cm^−1^ originating from C-O-C stretching in CA [39,57]. The peak at 1342 cm^−1^ originates only from PEO, and it is not affected by the addition of ZnO, rather being the broad band at 1366–1051 cm^−1^, which consists of two peaks, both originating from CA and PEO [39], that is affected by the addition of ZnO. Moreover, in the region between 1342 and 1368 cm^−1^ in samples with a higher ratio of CA component (Figure 8b), 1342 cm^−1^ is assigned to PEO and 1368 cm^−1^ assigned to CA (symmetric bending of CH_3_ [57]), and upon the addition of ZnO, the intensity of these peaks changes. In samples with a higher ratio of CA component (HNF samples, Figure 8b), the intensity of the peak around 1037 cm^−1^ decreases with the addition of ZnO, being the lowest in the sample with 2.5%.

As control samples, we compared the electrospun CA with ZnO and pure ZnO spectra (Figure 8c,d). As can be seen, the peaks corresponding to ZnO, at 873 cm^−1^ and 984 cm^−1^ (Figure 8d), are also found in the spectra of electrospun CA–ZnO (Figure 8c) as a small peak at 874 cm^−1^ and a small shoulder at 985 cm^−1^. In addition, the peaks at 1432 cm^−1^ (bending of CH_2_) and at 1664 cm^−1^ (hydrogen bonds [57]) in CA with ZnO become more prominent, confirming complex interactions and high affinity between CA and ZnO.

In the case of electrospinning, 12% CA solution was processed with ZnO (Figure 8c), while for blow spinning 12% CA solution, it was processed with PEO and ZnO (Figure 8b). According to the literature, preparation of pure 12% CA using SBS in our configuration of the device does not lead to formation of defect-free fibers [40], but processing CA with PEO leads to uninterrupted production of defect-free fibers [39].

These data lead to the conclusion that ZnO interacts with the CA component, confirmed by the fact that UV-vis spectroscopy did not detect significant release of ZnO into water upon dissolution of the PEO component. If ZnO nanoparticles are primarily found in the non-water-soluble CA rich phase, they will remain in that phase without leading to their release. Of course, it should not be overlooked that in the CA–PEO nanofiber composite, there is also a small amount of PEO incorporated into CA nanofibers as well [39], so even if some ZnO interacts with PEO through ether groups [58], the major portion of added ZnO is trapped inside CA nanofibers and moreover chemically interacts with CA, as FTIR analysis has shown.

It should be noted as well that strong electrostatic interactions must be present between CA and ZnO, since it is known that ZnO has a positive surface charge at neutral pH [59], while powdered CA has negative zeta potential at neutral pH [39], so this prevailing interaction between CA and ZnO is probably a consequence of strong electrostatic attraction that can exist during preparation of CA polymer solutions with nanoparticles. For future studies, the interactions between polymers that are used for nanofiber formations and nanoparticles could be assessed from zeta potential measurements.

## 4. Conclusions

In this paper, we report preparation of CA–PEO composite nanofibers with encapsulated ZnO nanoparticles. Study of the complex interactions between two polymers and nanoparticles during preparation of nanofibers revealed that the addition of ZnO did not influence the mean value of nanofiber diameter, but it did influence the size distribution and the mode (the most frequent value). Further, it was confirmed that addition of ZnO NPs into CA–PEO solution causes a reduction in viscosity when concentration of nanoparticles is low, i.e., when concentration is 2.5%, while with further increase in nanoparticle concentration, viscosity increases but remains lower than in the solutions without nanoparticles.

Release studies of ZnO from the composite nanofibers into water revealed that despite dissolving PEO in water, ZnO could not be detected with UV-vis spectroscopy, which confirmed that nanoparticle incorporation is induced by selectivity and ZnO mostly interacts with CA in composite CA–PEO. The ATR–FTIR analysis confirmed molecular interactions between the CA component in nanofibers and ZnO.

The results presented in this work elucidate a new path toward selective combination of more active agents to be incorporated into composite materials, where one active component could be attached to PEO and released immediately after contact with fluid, while inside the CA nanofibers, the remaining ZnO could provide prolonged activity. This could be especially useful for applications that require multifunctional materials.

The study in this article shows the possibility of processing a ternary composite, a system of two polymers with inorganic nanoparticles, using solution blow spinning. Since CA as polysaccharide cannot be processed with ZnO using SBS without the spinning aid of PEO (opposite to the relative ease of processing of CA–ZnO system with ES), this also presents a limitation in this study. Moreover, since PEO as spinning aid is soluble in water, future studies will aim at exploring other polymers to support processing of cellulose acetate with nanoparticles using solution blow spinning. In this way, multifunctional cellulose-based composite materials can be developed with solution blow spinning.

## Data Availability

The data presented in this study are available on request from the corresponding author.

## Supplementary Materials

The following supporting information can be downloaded at: https://www.mdpi.com/article/10.3390/polym16030341/s1, Figure S1: Comparison of Gaussian curves of samples produced using higher ratio of cellulose acetate in a mixture of CA/PEO (a) and lower ratio of cellulose acetate in a mixture of CA/PEO (b).
