# Supplementary material for: “Nano in Nano”—Incorporation of ZnO Nanoparticles into Cellulose Acetate–Poly(Ethylene Oxide) Composite Nanofibers Using Solution Blow Spinning
Source: Polymers (Basel). 2024 Jan 26;16(3):341. doi: 10.3390/polym16030341 (PMC10857684; doi:10.3390/polym16030341)
Supplement: Supplementary file 1 [file polymers-16-00341-s001.zip › polymers-2825368-supplementary.pdf]

-Supplementary material-

“Nano in Nano”—Incorporation of ZnO Nanoparticles into Cellulose Acetate–Poly(Ethylene Oxide) Composite Nanofibers Using Solution Blow Spinning

Caroline Voorhis [1,2], Javier González-Benito [2] and Ana Kramar [2,3,*]

1 School of Science, Marist College, 3399 North Road, Poughkeepsie, NY 12601, USA; caroline.voorhis1@marist.edu

2 Department of Materials Science and Engineering and Chemical Engineering, Institute of Chemistry and Materials Álvaro Alonso Barba, IQMAAB, Universidad Carlos III de Madrid, Avda. Universidad 30, 28911 Leganés, Spain; javid@ing.uc3m.es

3 Novel Materials and Nanotechnology Group, Institute of Agrochemistry and Food Technology (IATA), Spanish Council for Scientific Research (CSIC), Calle Catedrático Agustín Escardino Benlloch 7, 46980 Paterna, Spain

* Correspondence: akramar@iata.csic.es, akramar@ing.uc3m.es

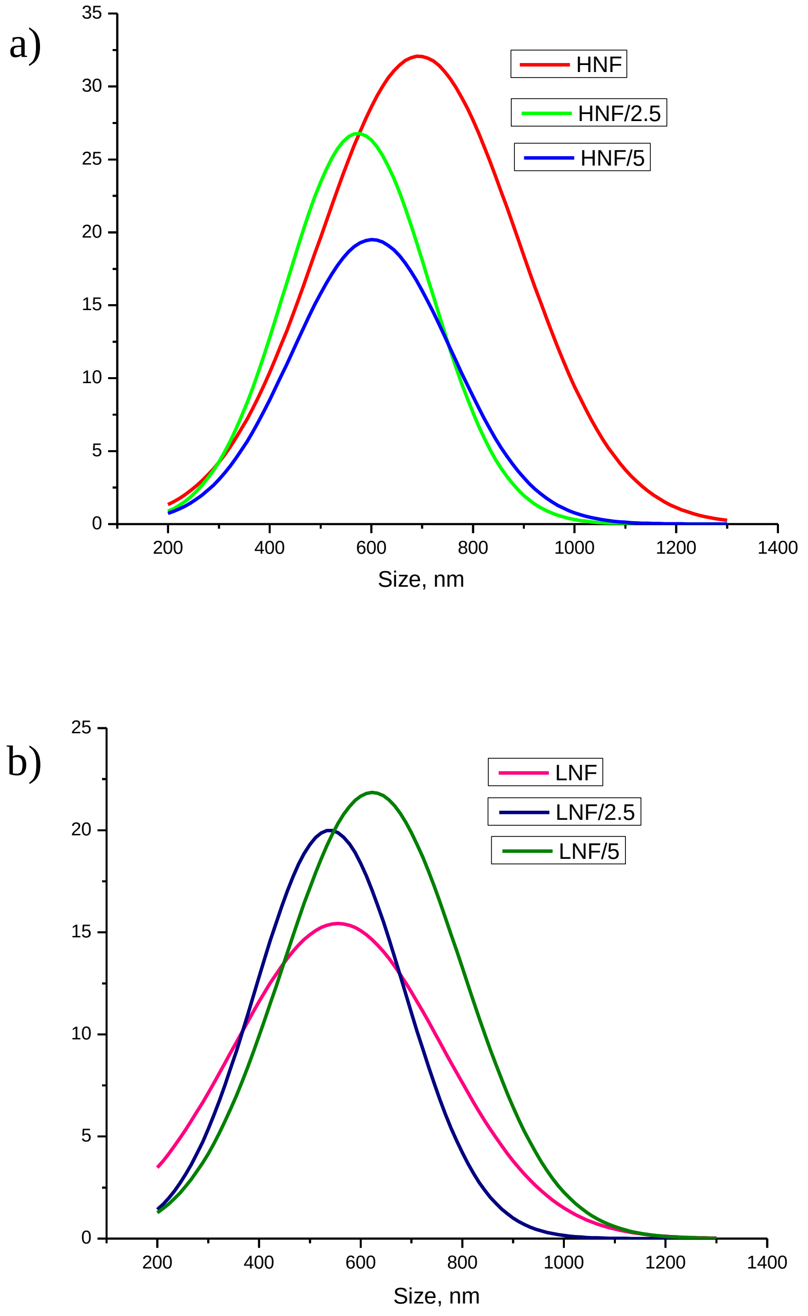

Figure S1. Comparison of Gaussian curves of samples produced using higher ratio of cellulose acetate in a mixture of CA/PEO (a) and lower ratio of cellulose acetate in a mixture of CA/PEO (b)
